# Scale dependent nanomechanical properties of dentin adhesive and adhesive-collagen composite

**DOI:** 10.3389/fdmed.2024.1423461

**Published:** 2024-11-28

**Authors:** Viraj Singh, Ranganathan Parthasarathy, Qiang Ye, Paulette Spencer, Anil Misra

**Affiliations:** ^1^ Institute for Bioengineering Research, University of Kansas, Lawrence, KS, United States; ^2^ Civil and Environmental Engineering Department, Tennessee State University, Nashville, TN, United States; ^3^ Department of Mechanical Engineering, University of Kansas, Lawrence, KS, United States; ^4^ Civil and Environmental Engineering Department, Florida International University, Miami, FL, United States

**Keywords:** collagen-adhesive composite, dentin adhesive, nanoindentation, nanoDMA, depth dependent, elastic modulus, viscoelastic

## Abstract

The complex micrometer construct at the interface that joins the composite material to the tooth surface in restorative dentistry is composed of the composite formed by infiltrating adhesive into demineralized dentin (collagen matrix). The overall performance of composite restorations is therefore directly linked to the properties of the polymerized adhesive and adhesive-collagen composite. Nanoindentation and nanoDMA tests are performed on model methacrylate based adhesive and collagen-adhesive composite to study their mechanical properties. The adhesive collagen composite is prepared by the infiltration of dentin adhesive into a completely demineralized bovine dentin. The obtained experimental results show that both the neat adhesive and the collagen-adhesive composite are heterogeneous materials at the spatial scales of property interrogation. It is also found that the reduced elastic modulus generally decreases with increasing indentation contact depth reaching an asymptote for both neat adhesive and collagen-adhesive composite. This reduced modulus behavior can be attributed to the increase in the indentation interaction volume. In addition, the measured frequency dependent storage and loss moduli indicate that both the neat adhesive and collagen adhesive composites are viscoelastic materials which are likely to exhibit creep deformation and rate-dependent behavior in physiological function.

## 1 Introduction

Adhesive biopolymers comprising hydrophobic/hydrophilic monomer pairs are often used as tissues adhesives (1, 2) or as dentin adhesives in restorative dentistry (3). The adhesive bio-monomer resin is expected to partly infiltrate the demineralized collagen matrices prior to polymerization. Thus, the adhesive and the adhesive-collagen composite are the two major material components of the resultant interfacial bond complex. These material components are of particular significance at the adhesive-dentin (*a*/*d*) interface which is formed when the relatively hydrophobic composite is joined to the subjacent tooth structure via dentin adhesive in cavity restoration. The mechanical integrity of dentin-adhesive interface is essential to the success of composite tooth restoration (3). The functional durability of this narrow interface is impacted by the complex bio-chemo-mechanical environment of the oral cavity that the interface experiences. Furthermore, the interface is a highly heterogeneous 3D construct of interdigitated materials from the viewpoint of composition and structure, which makes the characterization and mathematical descriptions difficult irrespective of the modeling scale. The characteristics of this interface also evolve with time as it is subjected to time and rate-dependent biomechanical loading while in function. All of these features lead to challenges not only for the laboratory analytical techniques, but also for mathematical modeling (4).

Clearly, the mechanical performance and durability or fatigue life of the tooth cavity restoration depends significantly upon the mechanical properties of the material components at the *a*/*d* interface (5–7). The *a*/*d* interface is arguably, the weakest link in composite tooth restorations (3, 8–10). The loss of integrity of this interface, even in cases in which the restoration remains nominally in-place, is clinically relevant because the micro-scale gaps will be infiltrated by enzymes, bacteria and oral fluids. The penetration of these agents into the spaces between the dentin and the composite can lead to recurrent caries, hypersensitivity, pulpal inflammation, and will eventually undermine the restoration. Irrespective of the mechanism by which the restoration fails, the dentin adhesive and collagen-adhesive composite plays a vital role in load transfer and maintenance of the mechanical integrity of the *a*/*d* interface (5, 11, 12).

Investigation of mechanical response of the *a*/*d* interface or other similar bio-interfaces using bond strength test seldom provides insight into the role played by each of the material components that make up the interface. On the other hand, a molecular-scale investigation of the physicochemical interaction between demineralized dentin collagen and the adhesive is highly non-trivial and involves considerable uncertainty. A more feasible approach is to investigate the interface under the assumption that mechanical interlock provides the needed structural integrity. Naturally, understanding the performance of individual material components is a pre-requisite for this approach. We have previously discussed the insights obtained from idealized unit cells representing mechanical interlock between adhesive and demineralized collagen (13). Clearly, there are intrinsic merits to understanding how the material components perform individually, as these insights can be used to better engineer the dentin-adhesive interface. Currently, limited and contradictory experimental results of a subset of mechanical properties, such as apparent elastic modulus, creep and relaxation times have been presented for demineralized dentin infiltrated with dentin adhesive (14–18). In our previous publications, we have reported on the millimeter scale mechanical behavior of the dentin adhesive and collagen adhesive composite using the 3 point bending tests (19–22). It is notable, however, that the *a*/*d* interfaces are typically a few micrometers in dimension, typically measuring ∼10 *μ*m and ranging from 1–15 *μ*m (11, 12). Therefore, it is important to examine the properties of polymerized adhesive and adhesive-collagen composite at the nano-meter scale as these are more relevant to the evaluation of the effect of *a*/*d* interface upon the mechanical response of composite restoration. In this work, we focus upon the scale and time-dependent nature of nanomechanical properties of the model methacrylate dentin adhesive and the collagen-adhesive composites under dry condition. The obtained results add to the work reported in (19–22) that use a model adhesive and collagen-adhesive composites formed using demineralized bovine dentin. The adhesive and collagen-adhesive composite samples are prepared under *in vitro* conditions. Nanoindentation quasi-static and nanoDMA tests are then performed at different contact depths and frequencies to obtain the scale and frequency dependent properties of these biopolymers. The results show that at the nano-meter scales polymerized adhesive and adhesive-collagen composite are spatially heterogeneous and viscoelastic rate-dependent materials. The spatial mechanical heterogeneity as well as temporal-dependency of these materials that are found at the *a*/*d* interface can have significant affect upon the long-term performance of composite restorations. The present study is performed in dry conditions to provide baseline information that is not confounded by complex effects induced by loading rates and the presence of moisture (20).

## 2 Materials and methods

### 2.1 Sample preparation

**Figure 1:**
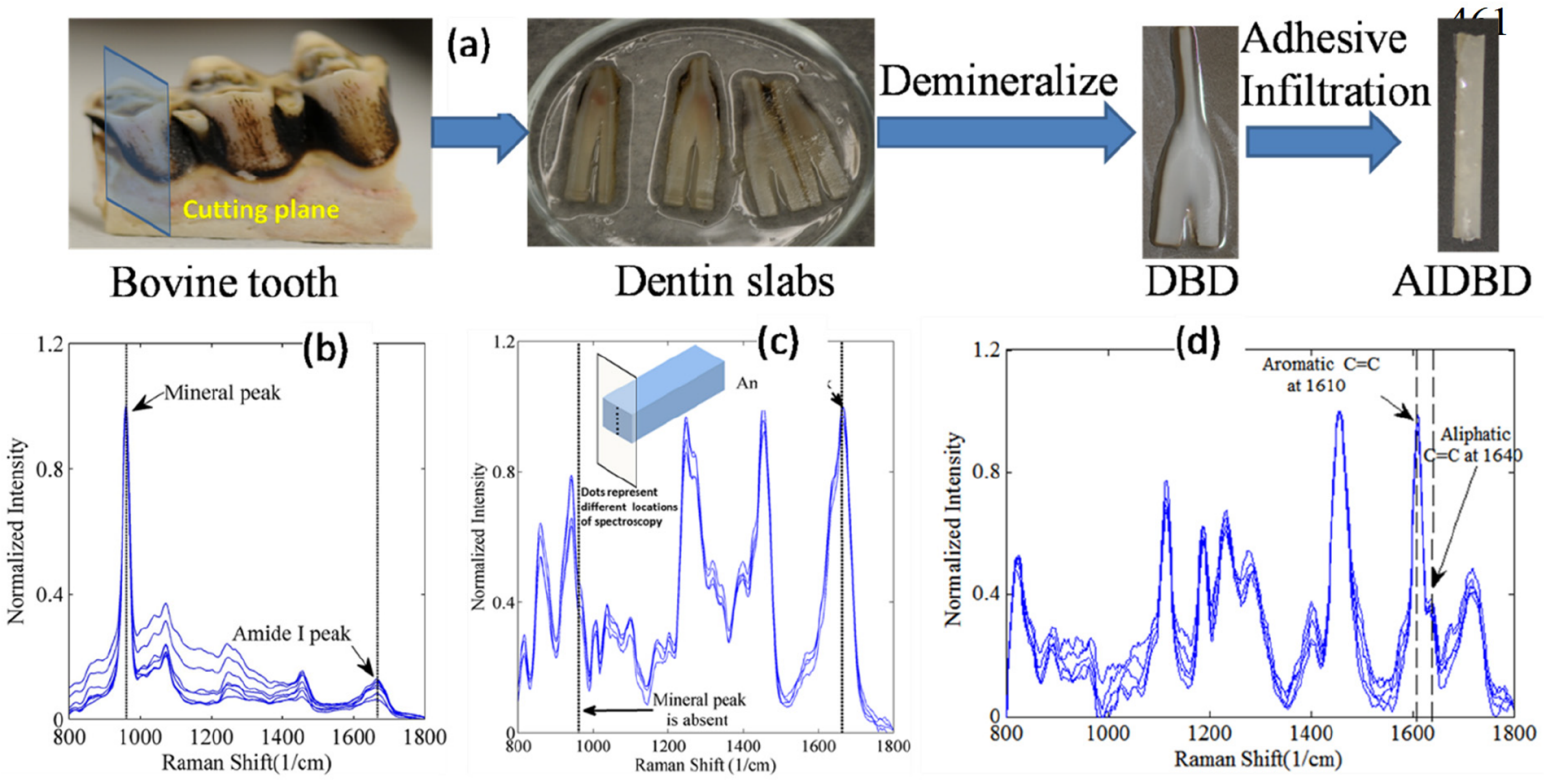
**(a)** AIDBD sample preparation. **(b)** Raman spectra of bovine dentin acquired at different locations before demineralization process. **(c)** Raman spectra of bovine dentin after the demineralization process acquired along the thickness for one randomly selected sacrificial sample as shown in the inset. **(d)** Raman spectra were acquired from points across the cross-section of one randomly selected sacrificial AIDBD sample to verify complete infiltration of dentin adhesive into DBD.

The sample preparation protocol followed in this work is the same as that described in (23) and has been included in this section briefly for completeness. Figure 1a shows the steps involved in adhesive-infiltrated demineralized bovine dentin (AIDBD) sample preparation. The recovered bovine dentin slabs were demineralized in 0.5M EDTA (pH 7.3) at 25°C for 10 days to obtain the demineralized bovine dentin samples (24). For determining that demineralization has been completed, Raman spectra were collected from the samples before (Figure 1b) and after (Figure 1c) 10 days of exposure to EDTA (19). In brief, LabRAM ARAMIS Raman spectrometer (LabRAM HORIBA Jobin Yvon, Edison, New Jersey) was used with a HeNe laser (*λ* = 633 nm, a laser power of 17 mW) as an excitation source. The instrument settings were as follows: 200 *μ*m confocal hole, 150 *μ*m wide entrance slit, 600 gr/mm grating, and 10x objective Olympus lens. Data processing was performed using LabSPEC 5 (HORIBA Jobin Yvon). The samples were mounted on a computer-controlled, high-precision *x*-*y* stage. The spectra of the samples were acquired over a range of 700–1,800 cm^−1^. These spectra were collected from 10 locations along an exposed section cut from a randomly selected sacrificial sample as shown in inset of Figure 1c. The absence of the mineral peak (P-O band at 960 cm^−1^) indicated complete demineralization (Figure 1c). Demineralized bovine dentin (DBD) slabs were kept in 70, 95% and 100% ethanol each for 12 h prior to dentin adhesive infiltration to gradually replace the water with ethanol. The model adhesive used consisted of 2-Hydroxyethylmethacrylate (HEMA, Acros Organics, NJ) and 2,2-bis[4-(2-hydroxy-3-methacryloxypropoxy) phenyl]-propane (BisGMA, Polysciences, Warrington, PA) with a mass ratio of 45/55 (HEMA/BisGMA). The following photoinitiators (all from Aldrich, Milwaukee, WI) were added to the adhesive: camphorquinone (CQ), ethyl-4-(dimethylamino) benzoate (EDMAB) and diphenyliodonium hexafluorophosphate (DPIHP). The amounts of photosensitizer, co-initiator amine and iodonium salt were fixed at 0.5 mass% with respect to the total amount of monomer. All the materials in this study were used as received.

The adhesive formulations were diluted with ethanol in 60/40 weight ratio. The DBD samples were then immersed in the adhesive ethanol mixtures and stored for 72 h in a dark room. The samples were then desiccated in a vacuum oven for 24 h to remove the solvent. After complete infiltration, adhesive-infiltrated demineralized bovine dentin (AIDBD) samples were polymerized using LED light curing unit of irradiance 250 mW/cm^2^ and area 6.25 mm^2^ for 40 sec (LED Curebox, Proto-tech, and Portland, OR, USA). The polymerized samples were stored in the dark at room temperature for 48 h to provide adequate time for post-cure polymerization. Adhesive infiltration was evaluated by collecting Raman spectra from the specimens (Figure 1d) from 10 locations along an exposed section cut from a randomly selected sacrificial sample. The AIDBD samples were stored for 72 h in a vacuum oven in the presence of a drying agent at 37°C to remove water that may have been absorbed during sample preparation. To obtain the smooth surface for the nanoindentation experiments, the polymerized sample of AIDBD were first polished using the 600 and 1,200 grit sandpaper. Thereafter, the final polishing was performed with polishing suspension with a particle size of 0.05 µm.

Dentin adhesive specimens using neat resin (NR) were prepared as disks of diameter 5 mm and height 2 mm by curing the liquid resin in an aluminum hermetic lid (900794.90, TA Instruments, New Castle, USA). To ensure oxygen inhibition and obtain smooth surface, a glass cover slip was placed on the lid during the polymerization process. Using this method, a specimen with a smooth surface was obtained. Finally, the degree of conversion (DC) of the AIDBD (collagen-adhesive composite) and neat resin samples was determined using Raman spectroscopy as described previously (23).

### 2.2 Nanoindentation instrument and data interpretation

*Quasi Static Tests*: Nanomechanical tests on NR and AIDBD specimens were performed using a DI Multimode V Atomic Force Microscope coupled with a Hysitron TS-75 Triboscope. A Berkovich diamond indenter with tip radius of 100 nm was used. In the current work, two different types of tests were performed. Test-A (fixed displacement), displacement was ramped to 400 nm at the rate of 40 nm/sec and then unloaded at the same rate to 0 nm. The reduced elastic modulus *E_r_* is computed from the unloading portion of the force displacement curve utilizing the included software with the indentation system [see also (25–27)]. Test-B (variable displacement), to study the effect of contact depth on the *E_r_*, which is another measure of spatial heterogeneity, displacement is ramped at the rate of 40 nm/sec to a specified displacement of *d* nm, and then unloaded at the same rate to half of the specified displacement, *d*/2 nm, to maintain contact. Subsequently, the targeted displacement i.e., *d* is increased, and the procedure is continued till the maximum displacement of 400 nm is reached. The reduced modulus is calculated from each unloading curve.

Before the beginning of the indentation procedure, both the NR and AIDBD specimens were scanned with small contact force to identify a 25 µm × 25 µm smooth region (RMS roughness ∼15 nm). Four such regions were identified, of which 2 were used for Test A and two for Test B. In each region, 25 indents were made at a spacing of ∼4 µm (4,000 nm) to ensure minimal interference of adjacent indent. Altogether 100 indents were performed for each material.

*NanoDMA Tests*: To study the frequency dependent properties of NR and AIDBD specimen nanoDMA tests were performed. These tests we performed at a static contact force of 120 µN, dynamic force of 3 µN, and frequencies ranging from 10 Hz–250 Hz. Similar to quasi-static nano-indentation tests both the NR and AIDBD specimens were scanned with small contact force to identify a 15 µm by 15 µm smooth region (RMS roughness ∼15 nm). Altogether, the nanoDMA test was performed at 25 locations each for the on NR and AIDBD samples.

## 3 Results

### 3.1 Raman spectroscopy and degree of conversion

Figures 1b–d presents the normalized Raman spectra of bovine dentin, demineralized bovine dentin and AIDBD. Raman spectra acquired after 10 days of demineralization, show absence of mineral peak (P-O band at 960 cm^−1^) and strong presence of amide I peak at 1,653 cm^−1^, this indicates complete demineralization of the bovine dentin slabs. The adhesive infiltration in the AIDBD samples was determined by acquiring Raman spectra across the cross-section of randomly selected samples as shown Figure 1d. The presence of spectral feature associated with the dentin adhesive (aliphatic C=C, peak at 1,640 cm^−1^ and the aromatic C=C at 1,610 cm^−1^) across the cross-section indicated complete infiltration. The interference of amide I peak at 1,653 cm^−1^ was removed while calculating the degree of conversion. The measured degree of conversion was 90.0% (±1.5%) and 87.0% (±0.5%) for the NR and AIDBD samples, respectively.

### 3.2 Nanoindentation tests

**Figure 2:**
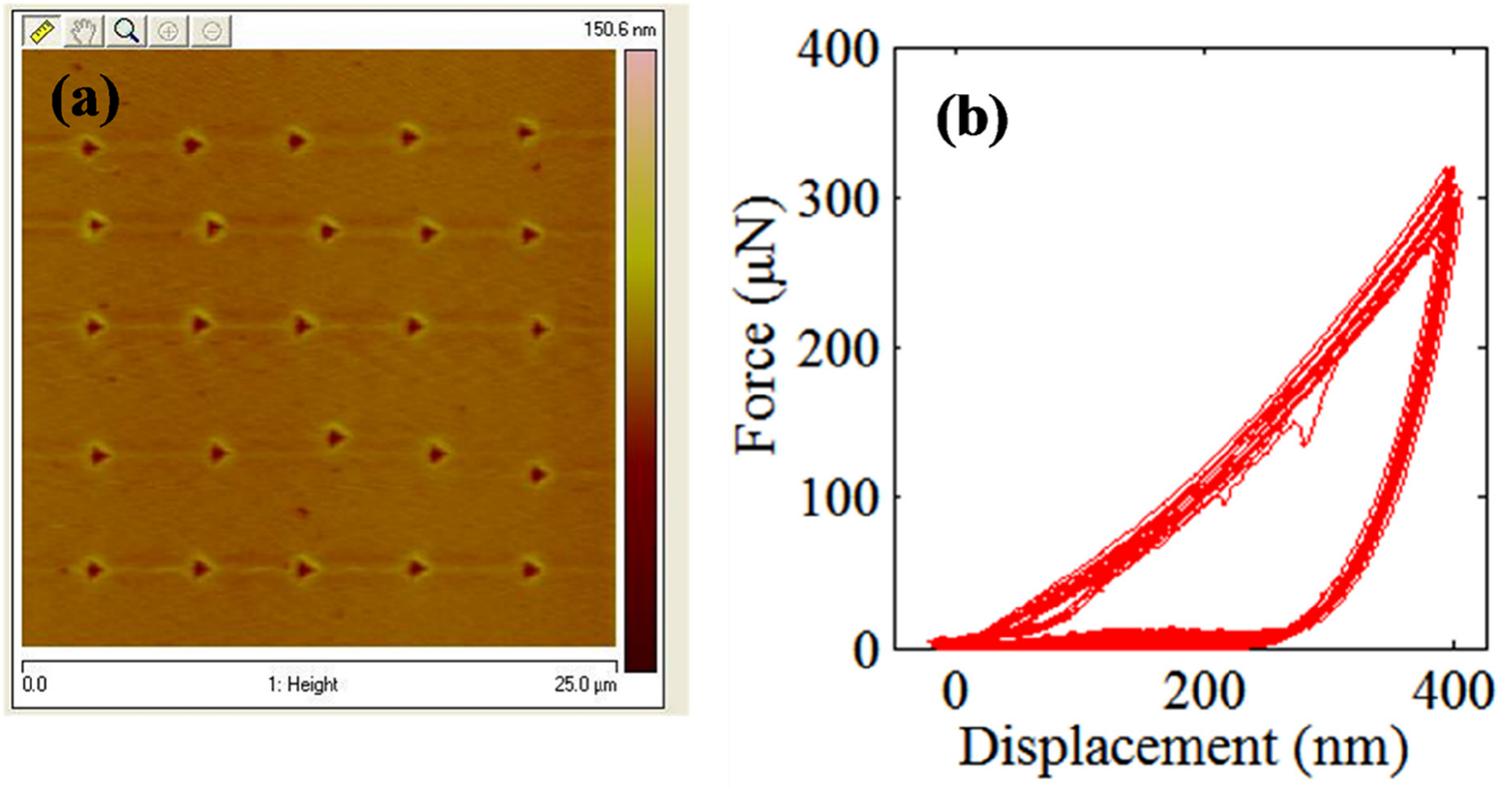
**(a)** topographical image showing 25 µm by 25 µm scan area with indentation location. **(b)** Force displacements curves at indents shown in Figure 2a.

**Figure 3:**
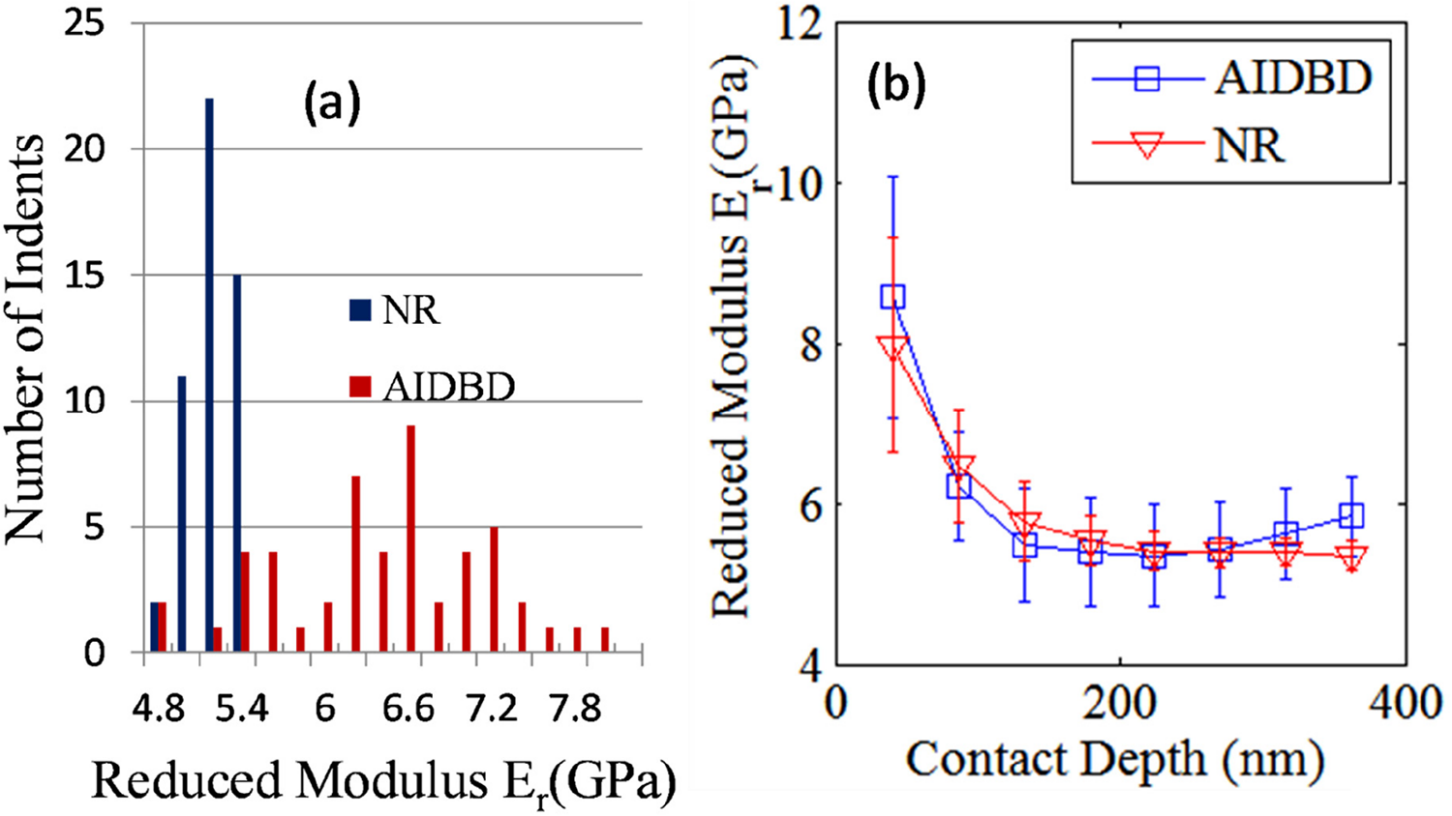
**(a)** histogram of reduced modulus for NR and AIDBD specimen. **(b)** Variation of reduced modulus with contact depth for NR and AIDBD specimen.

The results of the nanomechanical testing on AIDBD and NR specimens in dry condition are shown in Figures 2, 3. Figure 2a shows the representative raster pattern of a typical indent array. The indentation test was started from the top left corner of the 25 by 25 µm scan area. The measured loading-unloading force-displacement curves for this raster pattern of indents are shown in Figure 2b for the case of NR specimen which are analyzed using standard methods (25–27) to obtain the reduced modulus. It is noteworthy, that for AIDBD specimen, the slopes of the unloading curves show greater scatter. From the Type-A indentation tests (*n* = 50) we found the average (standard deviation) of the reduced modulus *E_r_* of 5.20 (±0.23) GPa, and 6.30 (±0.76) GPa, for the NR, and the AIDBD samples, respectively. A histogram of the reduced modulus is given in Figure 3a. From the histograms we observe that, NR resin have narrow range of reduced elastic modulus varying from 4.8–5.4 GPa compared to AIDBD which shows a much broader range from 4.8–8.0 GPa. The results of Type-B testing (*n* = 50) showing variation of reduced modulus *E_r_* with contact depth are given in Figure 3b. From Figure 3b we observe that, reduced modulus decreases with the contact depth and reaches nearly an asymptotic value at higher contact depths. At shallow depth of ∼40 nm, *E_r_* of 7.95 (±1.32) GPa and 8.57 (±1.51) GPa was found for NR and AIDBD, respectively. Whereas at the higher contact depth of ∼360 nm reduced modulus of 5.34 (±0.17) GPa and 5.83 (±0.50) GPa was obtained for NR and AIDBD, respectively.

**Figure 4:**
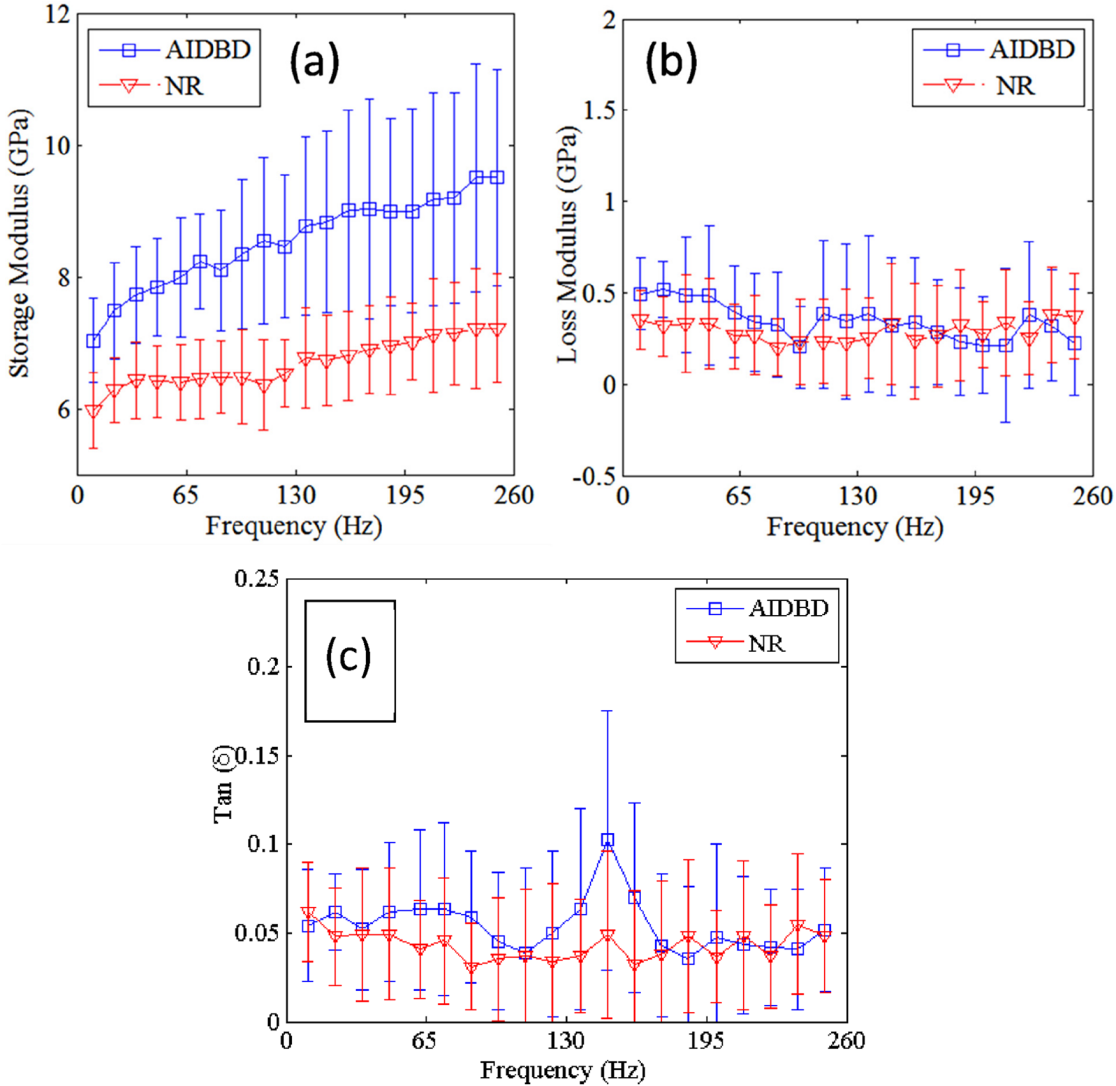
**(a)** storage, **(b)** loss moduli and **(c)** tan (δ) of AIDBD and NR sample as function of frequencies.

The result of nanoDMA tests on dentin adhesive and collagen adhesive composite is shown in Figure 4. Tests results show that, with the increase in frequency storage moduli of both NR and AIDBD increases. On the other hand, the loss moduli shown in Figure 4b do not exhibit any trend with the increase in frequency. From Figure 4a, we observe that NR and AIDBD have storage moduli of 5.0 GPa and 7.0 GPa, respectively, at frequency of 10 Hz, and 7.2 GPa and 9.5 GPa, respectively, at frequency of 250 Hz. At frequency of 10 Hz the loss moduli of NR and AIDBD were 0.35 GPa and 0.5 GPa, respectively, whereas at frequency of 250 Hz loss moduli were 0.375 GPa and 0.30 GPa for NR and AIDBD, respectively. From Figure 4c, we observe that NR and AIDBD have tan (δ) values of 0.062 and 0.054, respectively at frequency of 10 Hz, and at frequency of 250 Hz, both NR and AIDBD have tan(δ) of ∼0.048. The loss tangent for AIDBD shows a weak peak at about 140 Hz.

## 4 Discussion

The durability and biomechanical performance of restorations that utilize adhesive biopolymers, such as the composite tooth restoration, depends upon the mechanical properties of all the distinctly identifiable components in the so-called hybrid layer that forms the material interface between the adhesive and the tissue (5). Since these material components are present in small quantities, typically few micrometers in dimension, it is important to examine the properties of these components at the scale relevant to the application. In typical laboratory investigations available in the literature related to the *a*/*d* interfaces, the component material properties are explored for bulk specimen with dimensions of millimeter (14, 19, 20, 23). In the experimental investigations described in this paper, the effort has been made to characterize the nano-scale mechanical properties of dentin adhesive and ideal collagen adhesive composites, which are the two major components of *a*/*d* interface. Two types of mechanical experiments, quasi-static nano indentation and nanoDMA dynamic tests, were performed to obtain the scale-dependent and frequency-dependent mechanical properties of dentin adhesive and collagen adhesive composite. For both quasi-static and dynamic tests, all the indents were performed at the distance of 4 µm apart, which is ∼10 times the maximum contact depth to minimize the influence of neighboring indents on the mechanical response. It is noteworthy that quantitative interpretation methodologies of nano indentation and nanoDMA tests results have been evaluated recently for their accuracy (28, 29). The focus of the present work is to compare the results obtained with the same interpretation technique using tests done in the same conditions. Therefore, the comparison of properties with respect to spatial scales (which is correlated to indentation depth) and material types can therefore be considered as independent of the interpretation methodology.

From the results we observe that AIDBD have higher reduced elastic modulus compared to NR, which is consistent with our previous studies on millimeter-scale samples using 3-point bending tests (23) as well as the nanomechanical results reported in (16). Though AIDBD have higher reduced modulus, they also have higher standard deviation approximately 12% of the reported average *E_r_*, indicating larger heterogeneity compared to NR which has ∼4% standard deviation. This is expected because AIDBD is a composite of the dentin adhesive and demineralized bovine dentin primarily composed of cross-linked collagen fibrils arranged in some nano-scale structure reminiscent of that in the native dentin. Furthermore, the hydrophobic and hydrophilic components of the adhesive monomer may vary in their degree of penetration into wet demineralized dentin (30–32). The results of nano-indentation at varying depths helps to concur that in such nano-structured composites, heterogeneity can be expected even when the indentation depth is large enough to interrogate an estimated material volume in the order of 1 µm^3^ (estimated assuming that the bulb of influence of an indenter of diameter 100 nm is ∼500 nm, that is 5 times the diameter as predicted by). Further the effect of interaction scale on the nanomechanical properties is investigated by performing indents at different contact depths for NR and AIDBD sample. It is observed that, as the interaction volume or the contact depth increases, the reduced elastic modulus decreases till it reaches an asymptotic value. Such depth dependent reduced moduli have been observed for a number of materials including polymers (33–35) and has been attributed to a variety of mechanisms including surface and microstructural effects. Depth dependence also suggests that the material exhibits a granular or discrete nature with a characteristic length scale. In this case, indentation measurements at scales on the order of the characteristic length scale generally show a stiffer response as shown in our results. Interestingly, the standard deviation was also found to be larger at the shallow contact depths. For NR, the standard deviation was ∼16% and ∼3%, while for AIDBD it was ∼17% and ∼8% at contacts depth of 40 nm and 360 nm, respectively. The higher standard deviation indicates a larger degree of heterogeneity at small indentation depths, since the indenter is interacting with a smaller volume. Therefore, to obtain the so-called bulk properties a reasonably large indentation depth should be chosen, especially for material systems containing more than one material component such as adhesive collagen composite.

For the nanoDMA testing, a contact force of 120 µN was applied which resulted in a contact depth of ∼140 nm. This contact depth was chosen based upon the results of static tests (shown in Figure 3b) to minimize the scale effect on the frequency dependent properties of AIDBD and NR. The increase of storage modulus with frequency and small loss modulus indicates that these materials are viscoelastic solid as observed in tests for millimeter scale samples (23). The weak peaking of the AIDBD loss tangent and, to a lesser extent, of the NR loss tangent in the mid-range frequencies is also suggestive of higher viscous contributions in this frequency range, compared to the low frequencies (relaxed behavior) and the high frequencies (elasticity dominant behavior), considering the crosslinked polymeric structure of these materials. We also note that the nanoDMA tests results corroborate the fact that AIDBD is stiffer compared to NR in dry environments, due to the presence of the collagen fiber network. It is worthwhile to mention here that under wet conditions, both AIDBD and NR are expected to soften as water infiltrates due to hydrophilicity disrupting the molecular scale interactions. However, as we have discussed in the case of millimeter scale specimen (23), the AIDBD samples are likely to soften more due to the additional presence of hydrophilic collagen. It is also noteworthy that the mechanical response of these hydrophilic-hydrophobic polymeric constructs, whether pure adhesive or adhesive-collagen composites, are susceptible to anomalous mechanical response that depends upon moisture diffusion rate under sustained mechanical loads as discussed in relation to millimetric experiments in (20). Such moisture diffusion-rate and mechanical loading rates are also expected to affect nanomechanical behavior and should be subject of careful future investigations.

It is worth noting that relatively few previous studies have been conducted to obtain the nano-mechanical properties of resin infiltrated dentin. Recently (16) measured nano-mechanical behavior of resin-infiltrated dentin and neat dentin adhesive. These nano-mechanical tests were performed at very small indentation depth <10 nm at frequency of 100 Hz and reported a value of ∼4.0 (±0.3) GPa for adhesive infiltrated dentin and ∼3.5(±0.3) GPa for neat adhesive under dry condition with uncharacteristically small standard deviations. These standard deviations are at variance with those observed in our experiment, wherein the standard deviation is found to be 16%–17% at 40 nm indentation depth. In addition, we find that standard deviation as well as the average reduced modulus changes with the contact depth. Other investigators (17, 18) obtained the elastic moduli of adhesive infiltrated dentin samples using ultrasonic testing. Similar to our observations, they have reported the values of elastic moduli for their adhesive infiltrated dentin to be higher than that of the neat adhesive. These ultrasonic experiments were performed for saturated samples at high frequencies (5–10 MHz). Under these high frequencies a typically stiffer response is obtained for water saturated materials owing to the inability of unbound water to migrate under loading, leading to undrained conditions (22, 36). Therefore, the values reported by Yasuda et al. cannot be directly compared to the elastic modulus obtained from our experiments. It is also noteworthy that for measuring bulk properties under quasi-static conditions, 3 point bending tests have been used and have shown that although in dry conditions the AIDBD is stiffer than NR, in wet conditions the behavior is reversed (14, 23). It is clear, that these few experimental efforts have only examined a subset of mechanical behavior, and none appear to have investigated the depth or scale dependent mechanical properties of resin-infiltrated dentin. Furthermore, nanomechanical data interpretation is typically based upon the assumption that the material is linear elastic and homogeneous. The results of this paper and our previous studies have shown that both NR and AIDBD are complex, nonlinear, moisture-, scale- and rate-dependent (19, 20, 23). Materials with such mechanical behavior require a new physics-based mathematical model to describe the adhesive and adhesive-collagen composites in a comprehensive manner (22, 37–41).

Finally, it is noted that, in the current work the polymerized adhesive and adhesive-collagen composite specimens were made in ideal laboratory conditions, but the dentin-adhesive interface formed under clinical conditions is complex and is affected by demineralization process (42, 43), composition of dentin, adhesive phase separation (22, 24, 44) and the hydrophilicity adhesive components. Therefore, a systematic study is needed to determine how the overall performance of the restoration is impacted by the *a*/*d* interface constituents. To this end, the study reported here forms part of a larger effort to understand *a*/*d* interface through a combination of mathematical modeling and experimental characterization.

## 5 Summary and conclusions

Dentin adhesive and ideal collagen-adhesive composites formed by the infiltration of dentin adhesives into demineralized bovine dentin were tested for their nanomechanical properties under dry conditions. Nanoindentation and nanoDMA experiments were performed at various contact depths and frequencies to demonstrate the effect of heterogeneity upon the depth dependence of the reduced modulus and frequency dependence storage and loss moduli. We observe from quasi-static experiments that collagen-adhesive composites have higher reduced modulus and spatial heterogeneity as compared to neat resin samples. Also, experiment results show that the reduced modulus of both neat resin and collagen-adhesive composite specimens depends upon the indentation contact depth or scale. With the increase in interaction volume reduced modulus decreases and approaches a constant value representing bulk properties at the corresponding scale. This shows that these materials are complex heterogeneous polymers at all spatial scales. In addition, the small-scale storage modulus is found to exhibit an increasing trend with loading frequency confirming that these materials exhibit creep deformations and rate dependent mechanical behavior. The nanomechanical properties of such materials have been rarely examined. To this end the current paper provides additional scale and frequency dependent data at scales relevant to investigation of *a*/*d* interface behavior in composite restoration and to indicate the type of issues that likely affect the restoration performance.

## Data Availability

The original contributions presented in the study are included in the article/Supplementary Material, further inquiries can be directed to the corresponding author.

## References

[1] Reece TB Maxey TS Kron IL. A prospectus on tissue adhesives. Am J Surg. (2001) 182(2):S40–S4. 10.1016/S0002-9610(01)00742-5 11566476

[2] Singer AJ Thode HC Jr. A review of the literature on octylcyanoacrylate tissue adhesive. Am J Surg. (2004) 187(2):238–48. 10.1016/j.amjsurg.2003.11.017 14769312

[3] Spencer P Ye Q Park J Topp EM Misra A Marangos O Adhesive/dentin interface: the weak link in the composite restoration. Ann Biomed Eng. (2010) 38(6):1989–2003. 10.1007/s10439-010-9969-6 20195761 PMC2871971

[4] Misra A Singh V Parthasarathy R. Material-tissue interfacial phenomena: challenges in mathematical modeling. Mater Tissue Interfacial Phenom. (2017):253–64. 10.1016/B978-0-08-100330-5.00010-8

[5] Singh V Misra A Marangos O Park J Ye Q Kieweg SL Fatigue life prediction of dentin–adhesive interface using micromechanical stress analysis. Dent Mater. (2011) 27(9):e187–e95. 10.1016/j.dental.2011.05.010 21700326 PMC3658468

[6] Misra A Sarikaya R. Computational analysis of tensile damage and failure of mineralized tissue assisted with experimental observations. Proc Inst Mech Eng Part H. (2020) 234(3):289–98. 10.1177/0954411919870650 PMC7028502 31426717

[7] Sarikaya R Ye Q Song L Tamerler C Spencer P Misra A. Probing the mineralized tissue-adhesive interface for tensile nature and bond strength. J Mech Behav Biomed Mater. (2021) 120:104563. 10.1016/j.jmbbm.2021.104563 33940485 PMC8206037

[8] Spencer P Ye Q Misra A Bohaty BS Singh V Parthasarathy R Durable bonds at the adhesive/dentin interface: an impossible mission or simply a moving target? Braz Dent Sci. (2012) 15(1):4–18. 10.14295/bds.2012.v15i1.790 24855586 PMC4028112

[9] Kleverlaan CJ Feilzer AJ. Polymerization shrinkage and contraction stress of dental resin composites. Dent Mater. (2005) 21(12):1150–7. 10.1016/j.dental.2005.02.004 16040118

[10] Roulet JF. Benefits and disadvantages of tooth-coloured alternatives to amalgam. J Dent. (1997) 25:459–73. 10.1016/S0300-5712(96)00066-8 9604577

[11] Misra A Spencer P Marangos O Wang Y Katz JL. Micromechanical analysis of dentin/adhesive interface by the finite element method. J Biomed Mater Res Part B. (2004) 70B:56–65. 10.1002/jbm.b.30012 15199584 PMC3678287

[12] Misra A Spencer P Marangos O Wang Y Katz JL. Parametric study of the effect of phase anisotropy on the micromechanical behavior of dentin-adhesive interface. J R Soc Interface. (2005) 2:145–57. 10.1098/rsif.2005.0029 16849175 PMC1629071

[13] Singh V. Nonlinear Rate-Dependent Material Model with Damage and Plasticity from Granular Micromechanics Approach. (2014).

[14] Chiaraputt S Mai S Huffman BP Kapur R Agee KA Yiu CK Changes in resin-infiltrated dentin stiffness after water storage. J Dent Res. (2008) 87(7):655–60. 10.1177/154405910808700704 18573986

[15] Gu LS Huffman BP Arola DD Kim YK Mai S Elsalanty ME Changes in stiffness of resin-infiltrated demineralized dentin after remineralization by a bottom-up biomimetic approach. Acta Biomater. (2010) 6(4):1453–61. 10.1016/j.actbio.2009.10.052 19887126 PMC2830350

[16] Ryou H Pashley DH Tay FR Arola D. A characterization of the mechanical behavior of resin-infiltrated dentin using nanoscopic dynamic mechanical analysis. Dent Mater. (2013) 29(7):719–28. 10.1016/j.dental.2013.03.022 23639453 PMC3817502

[17] Yasuda G Inage H Kawamoto R Shimamura Y Takubo C Tamura Y Changes in elastic modulus of adhesive and adhesive-infiltrated dentin during storage in water. J Oral Sci. (2008) 50(4):481–6. 10.2334/josnusd.50.481 19106478

[18] Yasuda G Inage H Takamizawa T Kurokawa H Rikuta A Miyazaki M. Determination of elastic modulus of demineralized resin-infiltrated dentin by self-etch adhesives. Eur J Oral Sci. (2007) 115(1):87–91. 10.1111/j.1600-0722.2007.00425.x 17305722

[19] Singh V Misra A Marangos O Park J Ye Q Kieweg SL Viscoelastic and fatigue properties of model methacrylate-based dentin adhesives. J Biomed Mater Res Part B. (2010) 95B(2):283–90. 10.1002/jbm.b.31712 20848661 PMC3674959

[20] Singh V Misra A Parthasarathy R Ye Q Park J Spencer P. Mechanical properties of methacrylate-based model dentin adhesives: effect of loading rate and moisture exposure. J Biomed Mater Res Part B. (2013) 101(8):1437–43. 10.1002/jbm.b.32963 PMC4023510 23744598

[21] Parthasarathy R Misra A Park J Ye Q Spencer P. Diffusion coefficients of water and leachables in methacrylate-based crosslinked polymers using absorption experiments. J Mater Sci Mater Med. (2012) 23(5):1157–72. 10.1007/s10856-012-4595-5 22430592 PMC3361067

[22] Misra A Parthasarathy R Ye Q Singh V Spencer P. Swelling equilibrium of dentin adhesive polymer formed on the water adhesive phase boundary: experiments and micromechanical model. Acta Biomater. (2014) 10:330–42. 10.1016/j.actbio.2013.09.017 24076070 PMC3843361

[23] Singh V Misra A Parthasarathy R Ye Q Spencer P. Viscoelastic properties of collagen-adhesive composites under water-saturated and dry conditions. J Biomed Mater Res A. (2015) 103(2):646–57. 10.1002/jbm.a.35204 24753362 PMC4203711

[24] Wang Y Spencer P. Hybridization efficiency of the adhesive/dentin interface with wet bonding. J Dent Res. (2003) 82(2):141–5. 10.1177/154405910308200213 12562889

[25] Li X Bhushan B. A review of nanoindentation continuous stiffness measurement technique and its applications. Mater Charact. (2002) 48(1):11–36. 10.1016/S1044-5803(02)00192-4

[26] Gibson RF. A review of recent research on nanoindentation of polymer composites and their constituents. Compos Sci Technol. (2014) 105:51–65. 10.1016/j.compscitech.2014.09.016

[27] Díez-Pascual AM Gómez-Fatou MA Ania F Flores A. Nanoindentation in polymer nanocomposites. Prog Mater Sci. (2015) 67:1–94. 10.1016/j.pmatsci.2014.06.002

[28] Kontomaris S Malamou A Stylianou A. The Hertzian theory in AFM nanoindentation experiments regarding biological samples: overcoming limitations in data processing. Micron. (2022) 155:103228. 10.1016/j.micron.2022.103228 35124406

[29] Kontomaris S-V. The hertz model in AFM nanoindentation experiments: applications in biological samples and biomaterials. Micro Nanosyst. (2018) 10(1):11–22. 10.2174/1876402910666180426114700

[30] Hashimoto M Ohno H Endo K Kaga M Sano H Oguchi H. The effect of hybrid layer thickness on bond strength: demineralized dentin zone of the hybrid layer. Dent Mater. (2000) 16(6):406–11. 10.1016/S0109-5641(00)00035-X 10967189

[31] Hashimoto M Ohno H Kaga M Endo K Sano H Oguchi H. *In vivo* degradation of resin-dentin bonds in humans over 1–3 years. J Dent Res. (2000) 79(6):1385–91. 10.1177/00220345000790060601 10890717

[32] Zou Y Armstrong SR Jessop JL. Quantitative analysis of adhesive resin in the hybrid layer using Raman spectroscopy. J Biomed Mater Res Part A. (2010) 94A(1):288–97. 10.1002/jbm.a.32692 20186729 PMC2875550

[33] Briscoe BJ Fiori L Pelillo E. Nano-indentation of polymeric surfaces. J Phys D Appl Phys. (1998) 31(19):2395–405. 10.1088/0022-3727/31/19/006

[34] Nix WD Gao HJ. Indentation size effects in crystalline materials: a law for strain gradient plasticity. J Mech Phys Solids. (1998) 46(3):411–25. 10.1016/S0022-5096(97)00086-0

[35] Zhou J Hsiung LL. Depth-dependent mechanical properties of enamel by nanoindentation. J Biomed Mater Res A. (2007) 81A(1):66–74. 10.1002/jbm.a.31012 17109413

[36] Misra A Marangos O Parthasarathy R Spencer P. Micro-scale analysis of compositional and mechanical properties of dentin using homotopic measurements. In: Andreaus U Iacoviello D, editors. Biomedical Imaging and Computational Modeling in Biomechanics. 4. Springer Netherlands (2013). p. 131–41.

[37] Misra A Singh V. Thermomechanics-based nonlinear rate-dependent coupled damage-plasticity granular micromechanics model. Continuum Mech Thermodyn. (2015) 27(4-5):787–817. 10.1007/s00161-014-0360-y

[38] Barick MC Gaillard Y Lejeune A Amiot F Richard F. On the uniqueness of intrinsic viscoelastic properties of materials extracted from nanoindentation using FEMU. Int J Solids Struct. (2020) 202:929–46. 10.1016/j.ijsolstr.2020.03.015

[39] Chiravarambath S Simha NK Namani R Lewis JL. Poroviscoelastic Cartilage Properties in the Mouse from Indentation. (2009). 10.1115/1.3005199 19045920

[40] Oyen ML. Nanoindentation of hydrated materials and tissues. Curr Opin Solid State Mater Sci. (2015) 19(6):317–23. 10.1016/j.cossms.2015.03.001

[41] Taffetani M Griebel M Gastaldi D Klisch S., Vena P. Poroviscoelastic finite element model including continuous fiber distribution for the simulation of nanoindentation tests on articular cartilage. J Mech Behav Biomed Mater. (2014) 32:17–30. 10.1016/j.jmbbm.2013.12.003 24389384

[42] Marangos O Misra A Spencer P Katz JL. Scanning acoustic microscopy investigation of frequency-dependent reflectance of acid- etched human dentin using homotopic measurements. IEEE Trans Ultrason Ferroelectr Freq Control. (2011) 58(3):585–95. 10.1109/TUFFC.2011.1841 21429849 PMC3695421

[43] Wieliczka DM Spencer P Kruger MB. Raman Mapping of the dentin/adhesive interface. Appl Spectrosc. (1996) 50:1500–4. 10.1366/0003702963904584

[44] Spencer P Wang Y. Adhesive phase separation at the dentin interface under wet bonding conditions. J Biomed Mater Res. (2002) 62(3):447–56. 10.1002/jbm.10364 12209931

